# The Involvement of LvSRSF2 in Circular RNA Biogenesis and Its Role in Immunity Against White Spot Syndrome Virus (WSSV) in *Litopenaeus vannamei*

**DOI:** 10.3390/ijms26135981

**Published:** 2025-06-21

**Authors:** Wutthipat Potiyanadech, Cheeranan Sriphuttha, Tuangrak Seabkongseng, Neung Teaumroong, Panlada Tittabutr, Pakpoom Boonchuen

**Affiliations:** 1 Institute of Research and Development, Suranaree University of Technology, Nakhon Ratchasima 30000, Thailand; 2 School of Biotechnology, Institute of Agricultural Technology, Suranaree University of Technology, Nakhon Ratchasima 30000, Thailand

**Keywords:** white spot syndrome virus, *Litopenaeus vannamei*, serine/arginine splicing factors, circRNAs, immune response

## Abstract

Serine/arginine splicing factors (SRSFs) are critical regulators of gene expression that influence alternative splicing through RNA binding via the RNA recognition motif (RRM). Circular RNAs (circRNAs) are a subset of non-coding RNAs that exhibit differential expression in WSSV-infected *Litopenaeus vannamei*. This study investigates the role of LvSRSF2 in regulating circRNA expression in response to WSSV infection. LvSRSF2 was highly expressed in hemocytes and upregulated during WSSV infection. Silencing LvSRSF2 using dsRNA significantly upregulated the expression of circRNAs (circ-Alpha2, circ-Anillin, circ-Hemocytin, circ-Nephrin, and circ-Toll) in both WSSV-infected and uninfected shrimps at 72 h post-injection with dsRNAs. Knockdown of LvSRSF2 also significantly reduced WSSV copy numbers at 24 h post-infection and extended shrimp survival, with knockdown shrimp surviving up to 9 d compared to the control group. In addition, circ-Hemocytin, an SRSF2-related circRNA, was predicted to interact with six miRNAs targeting immune-related genes such as Toll, STAT, NF-κB, and Vago4. Following WSSV infection, circ-Hemocytin expression increased at 24 and 48 hpi, and the immune genes STAT and Vago4 were also upregulated, suggesting a potential circRNA–miRNA–mRNA regulatory axis in shrimp antiviral defense. Furthermore, targeted suppression of circ-Hemocytin expression using siRNAs significantly reduced its expression without affecting the corresponding linear transcript and resulted in a notable decrease in WSSV load in shrimp gills, highlighting its potential role in antiviral defense.

## 1. Introduction

White spot syndrome virus (WSSV) is a rod-shaped, double-stranded DNA virus from the family *Nimaviridae* and the genus *Whispovirus* that causes white spot disease (WSD) in shrimp [1,2]. Infected shrimp typically die within 3–5 d. The innate immune system, comprising cellular and humoral components, is the primary defense mechanism against infections in shrimp [3]. This system relies on pattern recognition receptors (PRRs) that detect pathogen-associated molecular patterns, triggering pathways that produce effector molecules to neutralize pathogens [3].

In addition, non-coding RNAs (ncRNAs) act as the primary protective agents against viral infections in this mechanism [3,4]. NcRNAs are classified into housekeeping ncRNAs, such as ribosomal RNA and transfer RNA, and regulatory ncRNAs, including small interfering RNAs (siRNAs), microRNAs (miRNAs), and circular RNAs (circRNAs) [5]. CircRNAs are ncRNAs with a closed-loop structure without 5′ and 3′ ends, and many studies have reported on their role in the immune systems of organisms [6,7]. CircRNAs have been discovered to influence viral infections by controlling the expression of genes, acting as molecular sponges for microRNAs, and regulating signaling pathways [8,9,10,11,12]. In humans, circRNAs like circ-ATP5H influence hepatitis B virus (HBV) replication and progression [10].

In the shrimp, circRNAs involved in virus infection were identified using transcriptome analysis [4,13]. A recent investigation examined the differentially expressed circRNAs (DECs) in WSSV-infected shrimp (*L. vannamei*) versus uninfected shrimp. The study identified 160 DECs that were upregulated and 130 DECs that were downregulated after WSSV infection [4]. In addition, a study on circRNAs involved in yellow head virus (YHV) infection found 177 upregulated circRNAs and 181 downregulated circRNAs in infected shrimp [13]. CircRNAs with deafferented expression have been found to include important genes related to the immune system, such as alpha-1-inhibitor-3, calpain-B, integrin-V, hemicentin-2, hemocytin, mucin-17, proPO2, and rab11-FIP4 [4]. These circRNAs are believed to participate in the antiviral response by regulating the expression of immune-related genes, acting as miRNA sponges, or modulating RNA-binding proteins. These findings highlight the significance of circRNAs in the shrimp’s response to viral infection.

Alpha-2-macroglobulin has been extensively studied for its role in the clotting system, where it inhibits fibrinolysis of the clotting complex [14]. It is also upregulated in response to WSSV infection [15]. Anillin was initially identified in *Drosophila* through actin filament affinity chromatography and immunofluorescence techniques [16]. Anillin is an actin-binding protein that plays a vital role in the progression of the mitotic cell cycle in metazoans [17]. Hemocytin plays diverse roles in the innate immunity of invertebrates. It functions as an adhesive protein and facilitates the capture of bacterial cells by promoting hemocyte aggregation [18,19]. During infection with *Nosema bombycis* in *Bombyx mori*, hemocytin is upregulated and binds to the external structure of *N. bombycis*, which leads to the agglutination of bacteria and hemocytes, followed by melanization [20]. Nephrin is a protein located in the podocyte pedicles and plays a critical role in ultrafiltration [21,22]. In crayfish, the epithelial cells of the coelomosac exhibit numerous pedicles that resemble vertebrate podocytes and are involved in urine filtration [23]. In addition, nephrin is an integral component of the slit diaphragm in podocytes and is essential for stabilizing this structure in vertebrate kidneys [24].

The innate immune response is typically triggered by recognizing pathogen-associated molecular patterns (PAMPs), which are conserved structures or motifs that are common to various classes of invading organisms. These patterns are detected by a diverse array of host PRRs [25]. Among the key PRRs is the Toll receptor superfamily, which includes invertebrate Tolls and vertebrate Toll-like receptors (TLRs). This receptor family is now recognized as a primary pathogen sensor across all metazoans [26]. In *L. vannamei*, three Toll receptors—Toll1, Toll2, and Toll3—are upregulated after the WSSV challenge, although their specific roles during WSSV infection remain poorly understood [27]. Studies on shrimp Tolls from *C. quadricarinatus*, *P. clarkii*, and *Macrobrachium rosenbergii* have demonstrated their ability to induce the expression of antimicrobial peptides (AMPs) in response to WSSV infection, and it is suggested that these AMPs may have antiviral functions [28,29,30]. Additionally, WSSV infection has been shown to activate numerous Toll pathway genes in *P. monodon*, and the entire pathway plays a crucial role in the immune response during infection [31]. A recent study revealed that Toll3 from *L. vannamei* can activate the expression of interferon regulatory factor (IRF) and its downstream effectors, Vago4 and Vago5. This highlights its potential role in host antiviral immunity through a mechanism that is independent of the canonical Toll signaling pathway [32].

Serine/arginine splicing factors (SRSFs) are essential components of RNA splicing and are involved in various RNA metabolic processes, including transcription, translation, and decay [32]. CircRNAs can interact with SRSFs to influence cellular and viral processes. For example, circular phospholipase C epsilon 1 (circPLCE1) binds with SRSF2 to regulate its expression, promoting colorectal cancer progression. Additionally, SRSFs also play roles in viral gene transcription and splicing [33]. For instance, SRSF1 binds to the human papillomavirus (HPV-16) splicing sites, and its reduced expression decreases viral mRNA splicing, thereby limiting HPV-16 proliferation [34,35]. Despite these findings, the role of SRSFs in virus-infected crustaceans remains unexplored.

However, studies have not examined the role of SRSFs in relation to virus infection in crustaceans. Therefore, the aim of this study was to determine whether the relation between SRSFs and circRNAs inhibits or promotes WSSV infection in the shrimp *L. vannamei*. The results contribute to the basic understanding of the key roles between circRNAs and SRSFs in response to WSSV infection in shrimp. In addition, the findings could lead to the discovery of biomarkers for detecting WSSV infection in shrimp and the development of methods to prevent or treat infections.

## 2. Results

### 2.1. Sequence and Phylogenetic Analysis of LvSRSFs

The search of shrimp sequences indicated that multiple isoforms of LvSRSFs have been reported in the NCBI database. Isoforms containing RRM domains were selected using SMART. The RRM is a frequently observed RNA-binding domain in proteins that plays key roles in RNA biogenesis, processing, transport, and degradation [36].

**Figure 1:**
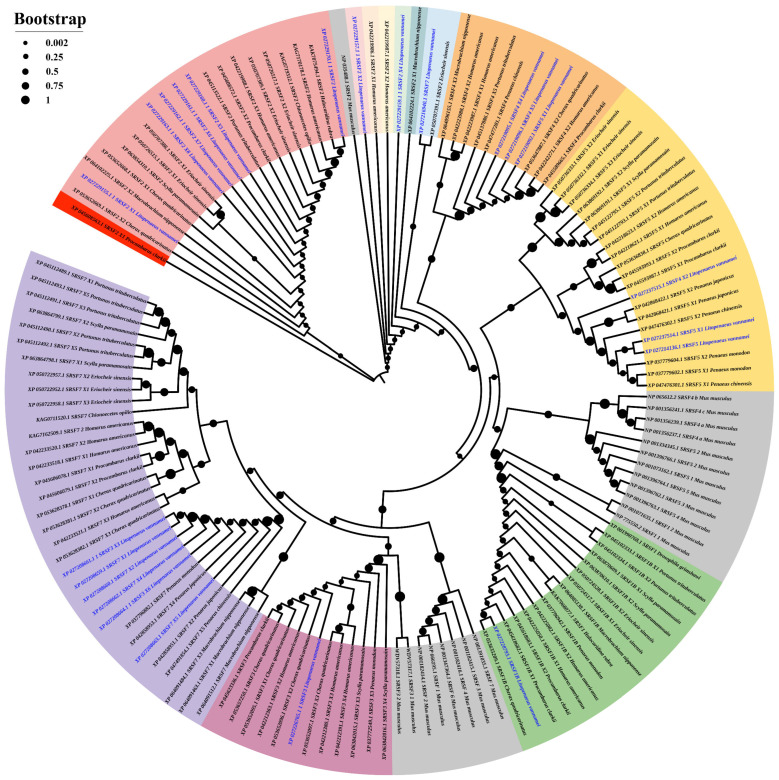
The phylogenetic analysis of SRSF proteins in white shrimp and other species. Using MEGA 11.0, a neighbor-joining phylogenetic tree was created for SRSF amino acid sequences from various organisms, employing 1000 bootstrap replicates, the Jones–Taylor–Thornton (JTT) model, and a gamma distribution with a shape parameter equal to 1 to evaluate node confidence. The pink-highlighted region represents Clade 1, The red-highlighted branch indicates the LvSRSF2 protein from *Litopenaeus vannamei*, the focus of this study. The grey-highlighted region includes vertebrate SRSF orthologs, suggesting evolutionary conservation across species. The orange-highlighted region represents Clade 2, the yellow-highlighted region represents Clade 3, the green-highlighted region represents Clade 4, the dusty pink-highlighted region represents Clade 5, and the lavender-highlighted region represents Clade 6. The blue-colored font denotes invertebrate species, helping to distinguish them from vertebrates within the tree.

**Figure 2:**
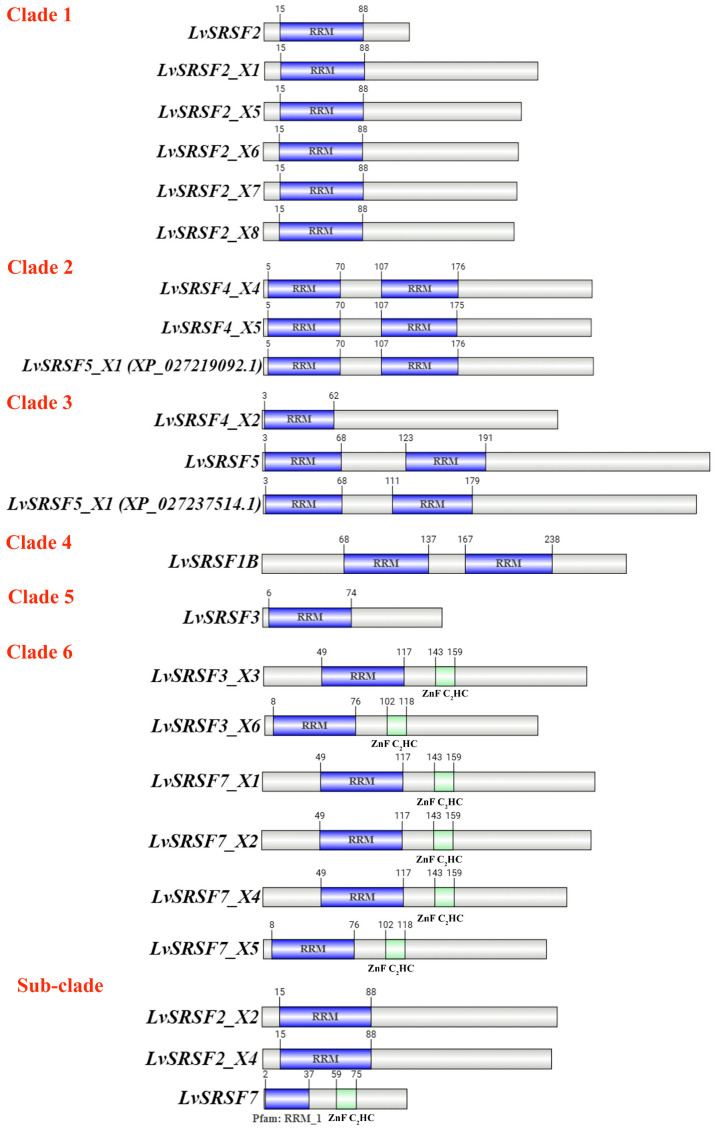
The diagram of RRM domain and Zn C_2_HC domain of SRSF protein in white shrimp. The blue box represents the RRM domain, while the green box indicates the ZnF C_2_HC domain.

SMART domain analysis identified multiple *L. vannamei* SRSF isoforms containing RNA recognition motifs (RRMs), including LvSRSF1B, LvSRSF2 (X1–X8), LvSRSF3, LvSRSF4 (X2, X4, X5), LvSRSF5, and LvSRSF7 (X1, X2, X4, X5). Phylogenetic analysis (Figure 1) revealed that LvSRSF1B clustered with SRSF1B from *Cherax quadricarinatus*, while LvSRSF2 isoforms were closely related to SRSF2 proteins from *Halocaridina rubra*, *Macrobrachium nipponense*, and *Portunus trituberculatus*. Notably, LvSRSF3 X3 and X6 grouped with SRSF7 isoforms from penaeid shrimp, supporting their reannotation as LvSRSF7 X3 and X6, respectively. In addition, LvSRSF4 X2 was more closely related to SRSF5 from penaeid shrimp, suggesting its reclassification as LvSRSF5 X2, whereas LvSRSF4 X4 and X5 clustered with SRSF4 isoforms from other crustaceans, supporting their annotation as LvSRSF4 X1. LvSRSF7 was found to be closely related to SRSF2 of *Eriocheir sinensis*, indicating possible annotation overlap. Domain architecture analysis (Figure 2) revealed three major structural patterns: isoforms such as LvSRSF1B, LvSRSF4 X4/X5, and LvSRSF5 contained two RRM domains; LvSRSF3 X3/X6 and LvSRSF7 isoforms had one RRM and one ZnF C2HC domain; and LvSRSF2 isoforms, LvSRSF3, and LvSRSF4 X2 featured a single RRM. These findings highlight the structural and evolutionary diversity of SRSF isoforms in *L. vannamei*, with potential implications for their functional specialization.

### 2.2. Tissue Distribution Analysis of LvSRSF Genes

In the tissue distribution experiment, the expression of each isoform of the LvSRSF gene was analyzed across seven tissues: the hemocytes, hepatopancreas, gills, muscle, stomach, heart, and intestines. Isoforms with significantly high expression in the gill or hemocytes were identified. The 16 primer pairs targeted the following isoforms: LvSRSF1B (XM_027372992.1), LvSRSF2 (XM_027373369), LvSRSF2 X1 (XM_027373354.1), LvSRSF2 X2 (XM_027373356.1), LvSRSF2 X4 (XM_027373358.1), LvSRSF2 X5 (XM_027373359.1), LvSRSF2 X6 (XM_027373360.1), LvSRSF2 X7 (XM_027373361.1), LvSRSF2 X8 (XM_027373362.1), LvSRSF3 (XM_027370964.1), LvSRSF4 (conserved isoforms; XM_027363291.1, XM_027363294.1, and XM_027363295.1), LvSRSF5 (XM_027358335.1), LvSRSF5 X1 (XM_027381713.1), LvSRSF5 X2 (XM_027381714.1), LvSRSF7 (conserved isoforms; XM_027352858.1, XM_027352859.1, XM_027352860.1, XM_027352861.1, XM_027352862.1, and XM_027352863.1), and LvSRSF7 (XM_027361139.1).

**Figure 3:**
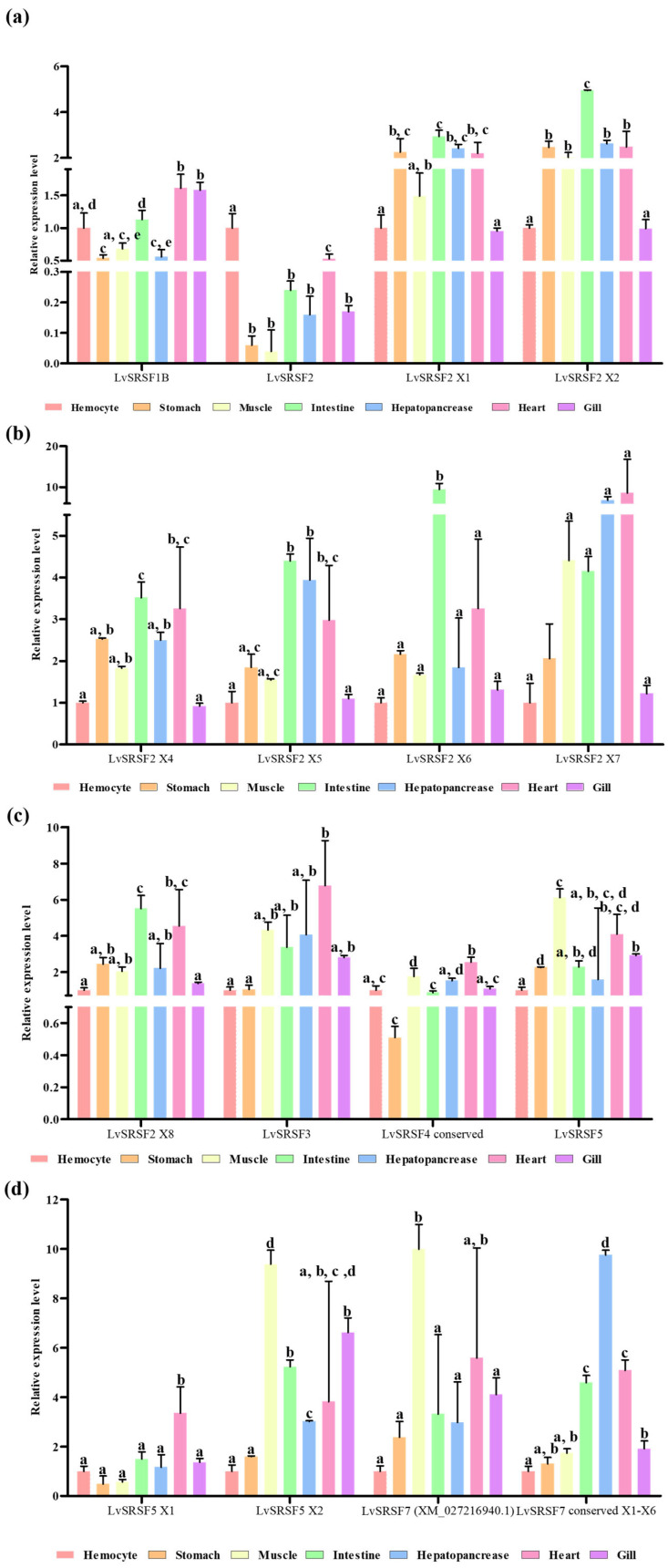
Expression levels of LvSRSFs gene. (**a**–**d**). Expression levels of LvSRSF1B, LvSRSF2, LvSRSF2 X1, LvSRSF2 X2, LvSRSF2 X4, LvSRSF2 X5, LvSRSF2 X6, LvSRSF2 X7, LvSRSF2 X8, LvSRSF3, LvSRSF4 conserved, LvSRSF5, LvSRSF5 X1, LvSRSF5 X2, LvSRSF7, and LvSRSF7 conserved in each tissue of white shrimp, respectively. Three replicates were used to assess the expression levels of all LvSRSF genes. Bars show the mean ± SD. A significant difference between sample groups (*p*-value < 0.05) is indicated by different letters (a, b, c, d, and e).

The tissue distribution analysis using qPCR for each LvSRSF isoform revealed that LvSRSF2 exhibited significantly higher expression in hemocytes compared to the other six tissues (*p*-value < 0.05) (Figure 3a). In contrast, the expression levels of other LvSRSF isoforms in the gills and hemocytes were not significantly different from those in other tissues (Figure 3). These findings suggest that LvSRSF2 may play a role in the immune system of the shrimp.

### 2.3. LvSRSF2 Expression Levels During WSSV Infection

**Figure 4:**
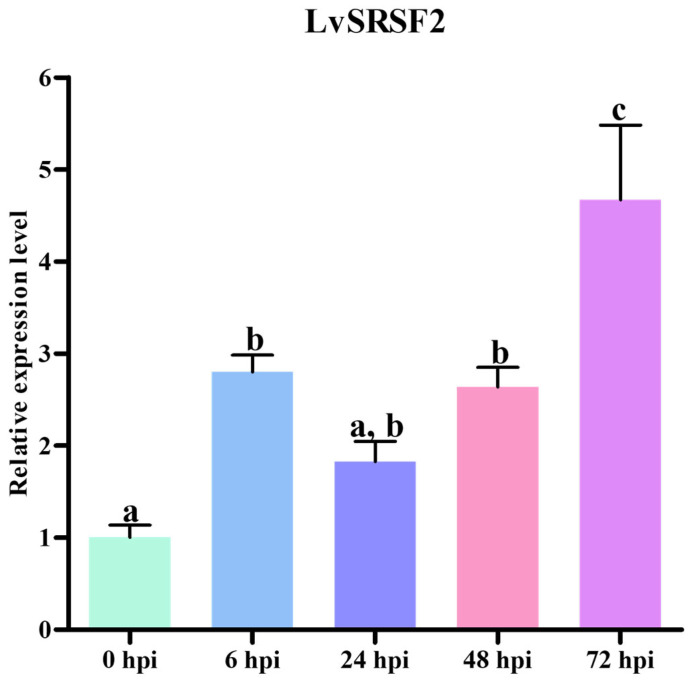
Expression levels of the LvSRSF2 gene at different times after WSSV infection in hemocyte tissue. Three replicates were used to assess the expression levels of the LvSRSF2 gene. Bars show the mean ± SD. A significant difference between sample groups (*p*-value < 0.05) is indicated by different letters (a, b, and c).

The expression analysis of LvSRSFs across seven tissues described in Section 2.2 revealed that LvSRSF2 was highly expressed in hemocytes compared to other tissues. Consequently, LvSRSF2 was further investigated to determine its expression levels in response to WSSV infection at different time points. The qPCR results showed that LvSRSF2 expression significantly increased at 6, 48, and 72 h post-WSSV infection compared to 0 h (*p*-value < 0.05) (Figure 4). Additionally, LvSRSF2 expression was, significantly, at its highest at 72 hpi compared to other time points (*p*-value < 0.05). These findings strongly suggest that LvSRSF2 plays a prominent role in responding to WSSV infection.

### 2.4. Effects of LvSRSF2 Knockdown on circRNA and liRNA Expression

The hypothesis that LvSRSF2 is involved in the expression of circRNAs following WSSV infection was investigated by analyzing the expression levels of circRNAs and liRNAs associated with the genes Alpha2, Anillin, Calpain, Hemocytin, Nephrin, and Toll. This analysis compared the results of LvSRSF2 knockdown to the control group (dsGFP) with and without WSSV infection.

**Figure 5:**
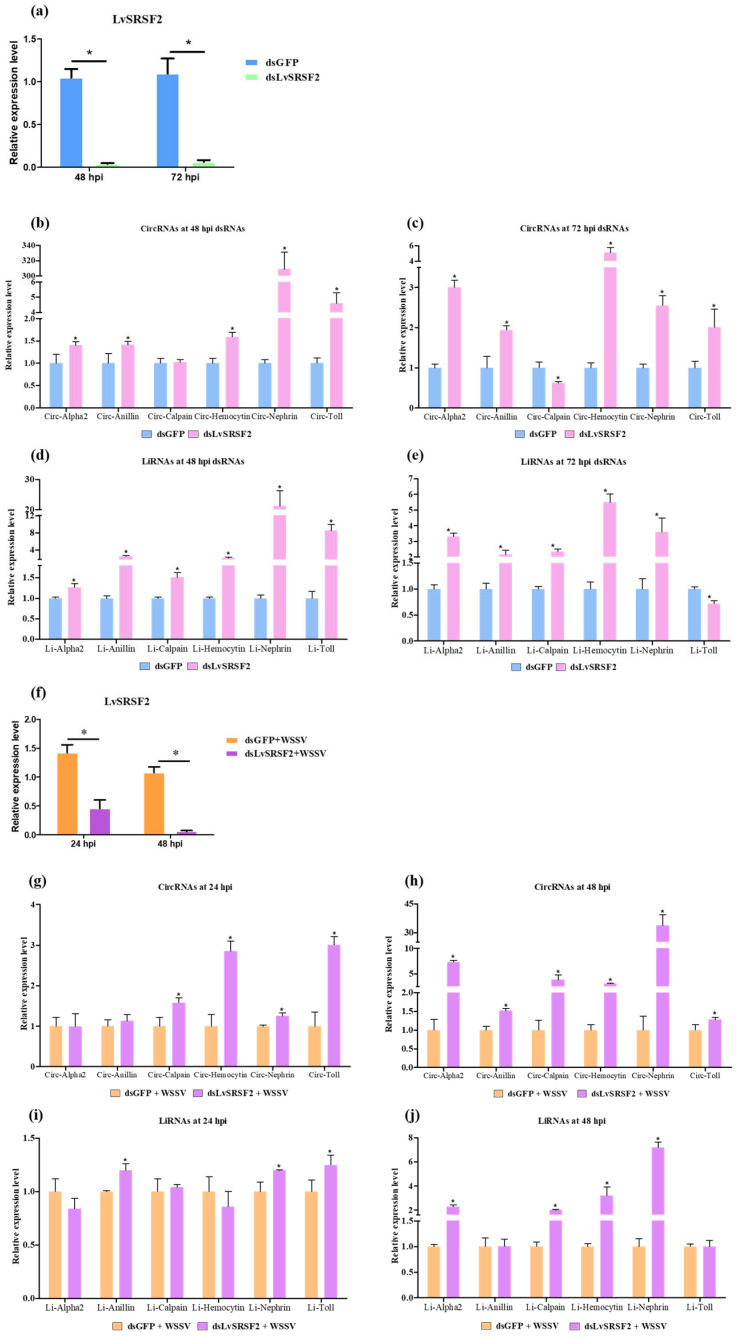
Expression levels of circRNAs and linear RNAs after knockdown LvSRSF2. (**a**) The expression of LvSRSF2 was determined at 48 and 72 hpi following injection of shrimp with dsGFP and dsLvSRSF2. (**b**,**c**) Expression levels of circRNAs following LvSRSF2 knockdown at 48- and 72-hpi with dsRNAs, respectively. (**d**,**e**) Expression levels of liRNAs following LvSRSF2 knockdown at 48 and 72 hpi with dsRNAs, respectively. (**f**) LvSRSF2 expression was analyzed at 24 and 48 hpi in shrimp injected with either dsGFP + WSSV or dsLvSRSF2 + WSSV. (**g**,**h**) Expression levels of circRNAs following LvSRSF2 knockdown and challenge with WSSV at 24 and 48 hpi with WSSV, respectively. (**i**,**j**) Expression levels of liRNAs following LvSRSF2 knockdown and challenge with WSSV at 24 and 48 hpi with WSSV, respectively. Bars show the mean ± SD. Asterisk indicate a significant expression level between sample groups at *p*-value < 0.05. All sample groups were performed by using three replicates.

The efficacy of dsLvSRSF2 in downregulating LvSRSF2 was evaluated by analyzing its expression profile at 48 and 72 hpi. The results showed that dsLvSRSF2 significantly suppressed LvSRSF2 expression, resulting in a 10-fold reduction compared to the controls (Figure 5a). At 48 hpi with dsRNAs, the qPCR results revealed that the circ-Alpha2, circ-Anillin, circ-Hemocytin, circ-Nephrin, and circ-Toll were significantly upregulated in the group where LvSRSF2 expression was knocked down using dsLvSRSF2 compared to the control group (*p*-value < 0.05). In contrast, the expression levels of circ-Calpain showed no significant difference compared to the controls (*p*-value < 0.05) (Figure 5b). At 72 hpi with dsRNAs, the expression patterns of circRNAs for each gene were consistent with those observed at 48 hpi with dsRNAs except for the circ-Calpain, which showed significantly decreased expression compared to the control group (*p*-value < 0.05) (Figure 5c). At 48 hpi with dsRNAs, the qPCR results showed that the li-Alpha2, li-Anillin, li-Calpain, li-Hemocytin, li-Nephrin, and li-Toll were significantly upregulated compared to the control group (*p*-value < 0.05) (Figure 5d). At 72 hpi with dsRNAs, the qPCR results were consistent with those at 48 hpi except for the li-Toll, which showed significantly reduced expression compared to the control group (*p*-value < 0.05) (Figure 5e).

The expression levels between WSSV-infected were compared following LvSRSF2 knockdown using samples from the WSSV-infected collected at 24 and 48 h post-WSSV infection (Figure 5f). WSSV was injected 24 h after the knockdown of LvSRSF2. The qPCR results at 24 h post-WSSV infection revealed that the circ-Calpain, circ-Hemocytin, circ-Nephrin, and circ-Toll were significantly upregulated compared to the control group (*p*-value < 0.05) (Figure 5g). At 48 h post-WSSV infection, the qPCR results showed that the circRNAs of all genes were significantly upregulated compared to the control group (*p*-value < 0.05) (Figure 5h).

For liRNAs, the qPCR results from WSSV-infected samples at 24 h post-WSSV infection showed that li-Anillin, li-Nephrin, and li-Toll were significantly upregulated compared to the control group (*p*-value < 0.05) (Figure 5i). In contrast, at 48 h post-WSSV infection, the qPCR results indicated that li-Alpha2, li-Calpain, li-Hemocytin, and li-Nephrin were significantly upregulated compared to the control group (*p*-value < 0.05) (Figure 5j). A comparative analysis between WSSV-infected and uninfected groups following the knockdown of LvSRSF2 revealed that silencing LvSRSF2 for 72 h after dsRNA injection significantly increased the expression of circ-Alpha2, circ-Anillin, circ-Hemocytin, circ-Nephrin, and circ-Toll in both groups (Figure 5c,h). These findings suggest that LvSRSF2 plays a role in suppressing the expression of these circRNAs in the shrimp.

### 2.5. Effects of LvSRSF2 Knockdown on WSSV Copy Number and Survival Rate Following WSSV Infection

**Figure 6:**
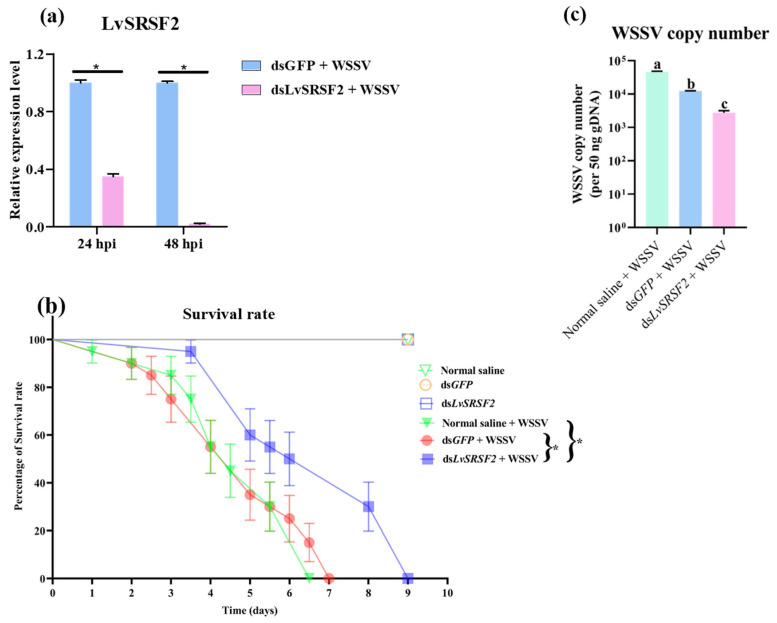
Effects of LvSRSF2 knockdown on WSSV copy number and survival rate in white shrimp following WSSV infection. (**a**) Expression levels of SRSF2 after knockdown SRSF2 and challenge WSSV at 24 and 48 hpi with WSSV. (**b**) Survival rate after knockdown with dsLvSRSF2 and challenge WSSV, and (**c**) WSSV copy number in gill sample (50 ng genomic DNA) of normal saline, dsGFP, and dsLvSRSF2 groups that were injected with WSSV at 24 hpi. Bars show the mean + SD. Different letters (a, b, c). In survival curve, asterisk indicates a significant difference in survival rate level between treatment groups using the log-rank at *p*-values < 0.05. The green inverted triangle with a line represents shrimp injected with normal saline. The orange circle with a line represents shrimp injected with dsGFP as a control group. The blue square with lines represents shrimp injected with dsLvSRSF2. The green-filled inverted triangle with a line represents shrimp injected with normal saline and subsequently challenged with WSSV. The red-filled circle with a line represents shrimp injected with dsGFP and subsequently challenged with WSSV. The blue-filled square with lines represents shrimp injected with dsLvSRSF2 and subsequently challenged with WSSV. Asterisk indicates a significant between sample groups at *p*-value < 0.05.

LvSRSF2 knockdown experiments were conducted to investigate the role of LvSRSF2 in the WSSV copy number and survival rate of WSSV-infected shrimp. Figure 6a showed that there was a significant reduction in LvSRSF2 expression in the knockdown group compared to the control group at 24 and 48 h post-WSSV injection. Furthermore, the survival analysis indicated that LvSRSF2 knockdown extended the survival time of WSSV-infected shrimp, with the knockdown group surviving up to 9 d, whereas the normal saline and dsGFP groups had maximum survival times of 6.5 and 7 d, respectively (Figure 6b). The analysis of WSSV copy number after LvSRSF2 knockdown and at 24 h post-WSSV injection revealed that the WSSV copy number was significantly lower in the LvSRSF2 knockdown group compared to the groups injected with normal saline or dsGFP (Figure 6c). These results suggest that silencing LvSRSF2 contributes to reducing WSSV replication and prolonging the survival of these shrimp following WSSV infection.

### 2.6. miRNA–circRNA Networks and Expression of Immune-Related circRNAs and Linear Counterparts

**Figure 7:**
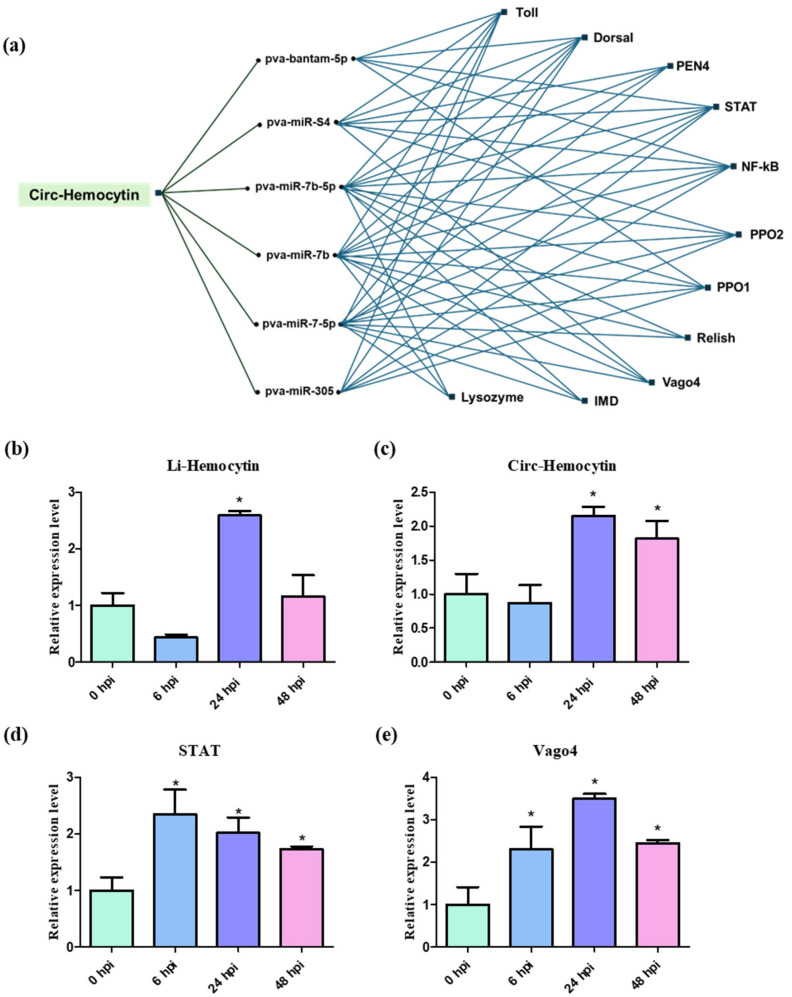
The miRNA–circRNA-mRNA interaction network (**a**), along with the expression levels of linear-Hemocytin (**b**), circ-Hemocytin (**c**), STAT (**d**), and Vago4 (**e**) in response to WSSV infection. Shrimp were intramuscularly injected with WSSV, and hemocytes were collected at 0, 6, 24, and 48 hpi. Gene expression was quantified by qRT-PCR, normalized to EF-1α, and presented as relative expression (mean ± S.D., n = 9). Asterisks indicate statistically significant differences compared to 0 hpi (*p*-values < 0.05). All experiments were performed in biological triplicates.

A previous study by Limkul et al. (2023) reported that one of the functional properties of circRNAs is their role as miRNA sponges for WSSV-responsive miRNAs, highlighting their regulatory function in modulating immune-related gene expression [4]. In this study, we selected an SRSF2-related circRNA, circ-Hemocytin, for further investigation. According to Limkul et al. (2023) [4], circ-Hemocytin was predicted to bind with six miRNAs: pva-bantam-5p, pva-miR-S4, pva-miR-7b-5p, pva-miR-7b, pva-miR-7-5p, and pva-miR-305. These miRNAs were subsequently used to predict potential immune-related target genes, including Toll, Dorsal, PEN, STAR, NF-κB, PPO1, PPO2, Relish, Vago4, IMD, and Lysozyme (Figure 7a). At 24 hpi, the expression of both li-Hemocytin and circ-Hemocytin was significantly upregulated, with circ-Hemocytin remaining elevated up to 48 hpi (Figure 7b,c). Based on this, we examined the expression patterns of selected miRNA-target genes following WSSV infection and found that STAR and Vago4 were upregulated at 6, 24, and 48 hpi, with fold changes ranging from 1 to 1.5 and 1.2 to 3.5, respectively (Figure 7d,e). These expression patterns are consistent with those of LvSRSF2, suggesting that LvSRSF2 may regulate the expression of immune target genes through circRNA–miRNA interactions.

### 2.7. Effects of siRNA Knockdown on circRNA and WSSV Copy Number

**Figure 8:**
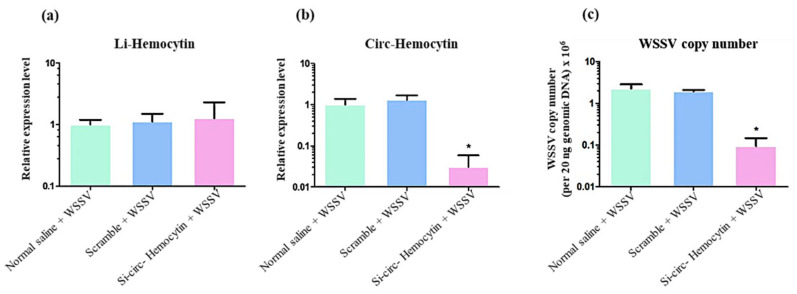
Effects of siRNA injection on circRNA expression and WSSV copy number. (**a**) Expression levels of linear Hemocytin (li-Hemocytin) at 48 hpi following si-circ-Hemocytin knockdown. (**b**) Expression levels of circ-Hemocytin after siRNA-mediated silencing. (**c**) Effect of circRNA silencing by siRNA on WSSV copy number. Results are presented as means ± SD (n = 9). Asterisks indicate statistically significant differences compared to other groups (*p*-values < 0.05).

The siRNA was specifically designed to target the back-splice junction of circ-Hemocytin. As shown in Figure 8a,b, the expression level of circ-Hemocytin was significantly reduced 50-fold in the group injected with si-circ-Hemocytin compared to both the NaCl and scrambled control groups. In contrast, the expression levels of li-Hemocytin remained unchanged across all experimental groups. Furthermore, the WSSV copy number in the gills of shrimp injected with si-circ-Hemocytin was significantly reduced 10-fold compared to all other groups (Figure 8c). These results suggest that LvSRSF2-related circ-Hemocytin might play a role in shrimp antiviral immunity; silencing circ-Hemocytin by siRNAs leads to decreased WSSV copy numbers, indicating the involvement of circRNA in modulates the innate immune response in shrimp.

## 3. Discussion

SRSFs are a family of RNA-binding proteins (RBPs) characterized by the presence of RNA RRM domains, which are essential for their function. Each RRM exhibits a slightly distinct consensus RNA-binding sequence, yet all recognize a short pyrimidine-rich motif and show a preference for sequences embedded within a longer pyrimidine tract that includes cytosines [37]. These domains enable SRSFs to bind specific RNA sequences, facilitating crucial RNA-processing events, including the biogenesis of circular RNAs (circRNAs) [38,39]. SRSFs with different numbers and types of RRM domains exhibit diverse functions in RNA binding and splicing regulation. Proteins like SRSF1 with two RRMs have complex roles in splicing specificity and RNA structure modulation [40,41,42,43], while those with a single RRM, such as SC35, rely heavily on their RS domains for localization and function [40].

Beyond their role in RNA processing, SRSFs have been increasingly recognized for their involvement in viral replication and host defense mechanisms. Among them, SRSF1 plays a pivotal role in the immune response, particularly in the function of CD8 T cells. It is essential for maintaining their proliferation and cytotoxic capabilities, as well as regulating key signaling pathways that support immune responses to viral infections in mice [44]. Additionally, SRSF1 acts as a cellular dependency factor regulated by interferons, influencing human immunodeficiency virus 1 (HIV-1) post-integration steps. While balanced levels of SRSF1 are crucial for efficient viral replication, its overexpression impairs HIV-1 transcription and splicing, whereas its knockdown increases viral RNA levels but disrupts the proportion of essential transcripts, such as HIV-1 viral infectivity factor (Vif) [45,46].

Similarly, SRSF2 is actively involved in modulating the viral life cycle. During herpes simplex virus type 1 (HSV-1) infection, SRSF2 is upregulated, influencing the transcription of HSV-1 genes. This suggests that SRSF2 functions as both a sensor and effector, regulating viral gene expression through epigenetic mechanisms [47]. Moreover, it serves as a transcriptional activator by binding to specific viral promoters, such as those of infected cell polypeptide 0 (ICP0), infected cell polypeptide 27 (ICP27), and thymidine kinase. Its role in splicing ICP0 pre-mRNA further highlights its dual function in transcriptional and post-transcriptional regulation of viral gene expression [48]. SRSF3 also plays a crucial role in viral pathogenesis, particularly in the HPV life cycle. It regulates the expression of viral capsid proteins under the control of the viral E2 protein, underscoring its potential as a target for antiviral therapies aimed at controlling HPV replication and infection [49]. Other SRSFs have distinct roles in regulating different viral infections. SRSF5 is essential for influenza A virus replication, as it enhances M mRNA splicing, a process critical for viral protein production. Inhibition of SRSF5 has been shown to significantly reduce virus replication, making it a potential antiviral target [50]. Conversely, SRSF9 has an inhibitory effect on HIV-1 production by inducing imbalanced mRNA splicing, thereby reducing viral infectivity [51]. SRSF10 is another key regulator of viral gene expression, with roles in multiple viruses. It is critical for the proper expression and processing of HIV-1 and other viral transcripts by influencing alternative splicing, a process essential for viral replication [52]. Additionally, SRSF10 acts as a restriction factor for HBV by regulating HBV RNA levels, particularly through its dephosphorylated form, which exerts an antiviral effect without altering RNA splicing. This highlights its potential as a therapeutic target for HBV treatment [53]. Furthermore, SRSF10 negatively affects the polymerase activity and replication of avian influenza virus by modulating the alternative splicing of chicken ANP32A, thereby altering the ratio of ANP32A transcripts. Interestingly, this regulatory effect is specific to avian influenza strains, with a weaker influence on mammalian-adapted influenza viruses [54]. However, the role of SRSF in shrimp has not yet been studied in response to viral infections.

In this study, we performed a phylogenetic tree analysis of LvSRSFs alongside SRSFs from crustaceans, *Drosophila grimshawi*, and *M. musculus* that possess RRM domains to investigate the evolutionary relationships of SRSFs in *L. vannamei*. The phylogenetic tree analysis revealed that SRSF in crustaceans can be classified into six major clades with distinct differences, along with seven additional sub-clades that were not grouped. Clade 1 consists of LvSRSF2, LvSRSF2 X1, LvSRSF2 X5, LvSRSF2 X6, LvSRSF2 X7, and LvSRSF2 X8, as well as SRSF2, SRSF2 X1, SRSF2 X2, and SRSF2 X3 from other crustaceans. This Clade 1 is highlighted in pink color in a tree (Figure 1). The phylogenetic tree also indicated that LvSRSF2 X5, LvSRSF2 X6, LvSRSF2 X7, and LvSRSF2 X8 were closely related. Additionally, Figure 2 showed that LvSRSF2, LvSRSF2 X1, LvSRSF2 X5, LvSRSF2 X6, LvSRSF2 X7, and LvSRSF2 X8 contained a single RRM domain located between amino acid positions 15–88 (Figure 2). The overall sizes of these LvSRSFs varied. However, although LvSRSF X2 and LvSRSF X4 were placed in separate sub-clades outside Clade 1, they contained the RRM domain in the same region of the protein as LvSRSF2, LvSRSF2 X1, LvSRSF2 X5, LvSRSF2 X6, LvSRSF2 X7, and LvSRSF2 X8 (Figure 2). Clade 2 consisted of LvSRSF4 X4, LvSRSF4 X5, and SRSF5 X1, as well as SRSF4, SRSF4 X1, SRSF4 X2, SRSF4 X3, and SRSF4 X5 from other crustaceans. Phylogenetic tree analysis showed that LvSRSF4 X4, LvSRSF4 X5, and SRSF5 X1 were closely related evolutionarily. In addition, Figure 2 also showed that LvSRSF4 X4, LvSRSF4 X5, and SRSF5 X1 (XP_027219092.1) contained two RRM domains. The first RRM domain was located at amino acid positions 5 to 70 in all three proteins. However, the second RRM domain of LvSRSF4 X4 and SRSF5 X1 (XP_027219092.1) was positioned between amino acids 107 and 176, while in SRSF4 X5, it was located between amino acids 107 and 175. Clade 3 consists of LvSRSF4 X2, LvSRSF5, and LvSRSF5 X1 (XP_027237514.1), along with SRSF5 from other crustacean groups. However, the phylogenetic tree also showed that LvSRSF5 and LvSRSF5 X1 (XP_027237514.1) were more closely related evolutionarily than LvSRSF4 X2. Figure 2 showed that LvSRSF5 and LvSRSF5 X1 (XP_027237514.1) both contained two RRM domains. The first RRM domain of LvSRSF5 and LvSRSF5 X1 (XP_027237514.1) was located between amino acid positions 3 to 68 in both proteins. However, the second RRM domain was located between amino acid positions 123–191 in LvSRSF5 and 111–179 in LvSRSF5 X1 (XP_027237514.1), respectively. Meanwhile, LvSRSF4 X2, which evolved further from LvSRSF5 and LvSRSF5 X1 (XP_027237514.1), contained a single RRM domain located between amino acids 3–62 (Figure 2). Clade 4 consists of LvSRSF1B, as well as SRSF1B and SRSF1 from *D. grimshawi* and other crustacean groups. It was found that LvSRSF1B contained two RRM domains, with the first located at amino acids 68–137 and the second at amino acids 167–238. Clade 5 consists of LvSRSF3 along with SRSF3, SRSF3 X1, SRSF3 X2, SRSF3 X3, and SRSF3 X4 from other crustaceans. LvSRSF3 contains one RRM domain, spanning amino acids 6–74. Clade 6 consists of LvSRSF3 X3, LvSRSF3 X6, LvSRSF7 X1, LvSRSF7 X2, LvSRSF7 X4, and LvSRSF7, as well as SRSF7, SRSF7 X1, SRSF7 X2, SRSF7 X3, and SRSF7 X5 from other crustaceans. Figure 2 showed that LvSRSF3 X3, LvSRSF3 X6, LvSRSF7 X1, LvSRSF7 X2, LvSRSF7 X4, and LvSRSF7 each contained one RRM domain and one ZnF C_2_HC domain, both of which were also found in LvSRSF7 outside of clade 6. The ZnF C_2_HC is integral to the function of Ring Finger Protein 125 (RNF125). It provides structural stability, facilitates interactions with ubiquitin-conjugating enzymes (E2), and ensures the enzyme’s activity through specific intramolecular contacts [42]. Additionally, Figure 2 showed that LvSRSF3_X3, LvSRSF7 X1, LvSRSF7 X2, and LvSRSF7 X4 each contained RRM and ZnF C_2_HC domains at the same positions. The RRM domain spanned amino acids 49–117, while the ZnF C_2_HC domain spanned amino acids 143–159. Although these LvSRSF proteins shared the same domains, the total protein lengths differed. LvSRSF3 X6 and LvSRSF7 X5 each contained RRM and ZnF C_2_HC domains at the same positions. The RRM domain spanned amino acids 8–76, while the ZnF C_2_HC domain spanned amino acids 102–118. Although these LvSRSF proteins shared the same domains, the total protein lengths differed. LvSRSF7 is a small SRSF that consists of an RRM and a ZnF C_2_HC domain located at amino acid positions 2–37 and 59–75, respectively. The phylogenetic tree analysis and the prediction of RRM domain positions are valuable for classifying LvSRSF and can contribute to predicting the functions of LvSRSF in each clade in the future.

Serine/arginine-rich splicing factors (SRSFs) comprise a family of twelve RNA-binding proteins that play essential roles in precursor messenger RNA (pre-mRNA) splicing by regulating splice site recognition and spliceosome assembly [55]. Several human glioblastoma studies have reported that specific circRNAs, such as circSMARCA5, act as sponges for SRSF family proteins, thereby modulating their regulatory functions in RNA splicing [56]. This study demonstrates that the injection of recombinant LvSRSF2 in shrimp leads to the upregulation of highly expressed circRNAs, suggesting that LvSRSF2 may function as an RNA-binding protein (RBP) that actively promotes the back-splicing process essential for circRNA biogenesis. Moreover, the expression of LvSRSF2 was significantly upregulated following WSSV infection, with transcript levels progressively increasing over time and peaking at 72 hpi, further supporting its potential role in the antiviral response of shrimp (Figure 4).

Notably, SRSF2, SRSF3, SRSF9, and SRSF10 were also found to be highly expressed in human tumors, implicating them as potential contributors to tumorigenic processes through their roles in RNA splicing regulation [57]. Silencing of LvSRSF2 resulted in a marked decrease in WSSV-responsive circRNAs, indicating that LvSRSF2 plays a central role in circRNA biogenesis during viral infection. In addition, the loss of LvSRSF2 function significantly increased the shrimp’s susceptibility to WSSV, as evidenced by elevated viral loads and mortality rates. Collectively, these findings demonstrate that LvSRSF2 is essential not only for circRNA formation but also for initiating an effective antiviral response, highlighting its critical role in the host’s defense mechanisms against WSSV.

A previous study by Limkul et al. (2023) demonstrated that circRNAs can function as miRNA sponges for WSSV-responsive miRNAs, thereby modulating the expression of immune-related genes and contributing to the host’s antiviral defense mechanisms [4]. Such sponge-like interactions between circRNAs and miRNAs can profoundly influence the progression of viral infections, such as WSSV in shrimp, by regulating the availability of immune-related miRNAs [58]. Circ-Hemocytin was predicted to interact with six miRNAs: pva-bantam-5p, pva-miR-S4, pva-miRb-5p, pva-miR-7b, pva-miR-7-5p, and pva-miR-305, all of which are associated with putative immune-related target genes such as Toll, Dorsal, PEN4, STAT, NF-κB, PPO1, PPO2, Relish, Vago4, IMD, and Lysozyme. Among these, STAT and Vago4 exhibited increased expression following WSSV infection, showing a pattern consistent with SRSF2 upregulation, which suggests a potential regulatory link between SRSF2, circ-Hemocytin, and antiviral immune signaling pathways. Previous studies have established that the JAK/STAT signaling pathway is commonly involved in antiviral defense, with shrimp STAT being activated upon WSSV infection [59]. Additionally, Vago functions as an interferon-like protein that connects interferon regulatory factors to immune gene expression, thereby supporting antiviral and antimicrobial immunity in shrimp [60]. These findings indicate that LvSRSF2 downregulation impairs circRNA biogenesis, facilitates WSSV replication, and compromises shrimp survival.

Within the context of viral circRNAs, circRNA-encoded proteins have been demonstrated to perform diverse functional roles, particularly in mediating antiviral responses. Previous studies have also identified the Quaking RNA-binding protein (QKI) as a critical regulator of circRNA biogenesis. In shrimp, LvQKI functions as a key factor in both circRNA formation and immune defense, underscoring the functional interplay between LvQKI and circRNAs in coordinating antiviral responses [61]. Furthermore, a circular RNA-encoded VP28 (ceVP28) has been shown to reduce WSSV replication and shrimp mortality by competitively binding to the host receptor Rab7 [62]. In the present study, the observed reduction in WSSV replication following circ-Hemocytin knockdown suggests that this circRNA may modulate the shrimp’s antiviral response (Figure 8), potentially acting as a competing endogenous RNA (ceRNA) that sequesters miRNAs involved in antiviral defense. Given that Hemocytin is a gene associated with immune function in invertebrates, its circular transcript may function distinctly from its linear counterpart, possibly regulating host–virus interactions at the post-transcriptional level.

Taken together, these results expand the understanding of circRNA-mediated regulation in crustacean immunity and provide the first evidence that targeting a specific circRNA can alter the outcome of viral infection in shrimp. Future studies should aim to elucidate the molecular mechanisms by which circ-Hemocytin modulates host–virus dynamics and identify its downstream targets, such as miRNAs or proteins involved in immune signaling. This knowledge could facilitate the development of novel RNA-based therapeutics.

Our results further indicate that the introduction of LvSRSF2 enhances circRNA expression, thereby strengthening the antiviral response in shrimp. This activation of the circRNA pathway likely contributes to the host’s defense against WSSV infection and underscores the regulatory role of LvSRSF2 in antiviral immunity. Consistent with the observed increase in survival rates following the WSSV challenge, these findings reinforce the pivotal function of LvSRSF2 in promoting circRNA-mediated resistance to viral infection.

## 4. Materials and Methods

### 4.1. Ethics Statement

The animal experiments adhered to the Ethical Principles and Guidelines for Scientific Animal Use, established by the National Research Council of Thailand. The Institute of the Suranaree University of Technology approved the animal use Protocol Number IACUC-66-17. In addition, the Institutional Biosafety Committee at Suranaree University of Technology (IBC-66-09) reviewed and approved the biosecurity considerations for this study.

### 4.2. Shrimp Samples

Healthy white shrimps (*L. vannamei*) were purchased from a commercial farm located in Chachoengsao Province, Thailand. The shrimps were cultivated in water with a salinity of 20 parts per thousand (ppt) and a temperature of 30 ± 2 °C. The shrimps were fed with commercial pellets twice daily. Shrimps with body length of approximately 9.5–10 cm and weight of approximately 5–7 g were used for quantitative real-time PCR (qPCR) for the determination of SRSFs in response to WSSV infection. WSSV challenge was performed by injecting shrimp intramuscularly with a 100 µL solution diluted to a concentration of 10^–5^. In contrast, the WSSV challenge of the survival experiment was performed by injecting shrimp intramuscularly with a 20 µL solution diluted to a concentration of 5×10^–5^. The concentration and amount of WSSV used were tested to be able to kill all normal shrimp within 7 days. These solutions were prepared from a filtered WSSV suspension with a viral copy concentration of 9.2 × 10^7^ copy per µL according to the method described by Xie et al., 2005 [63]. Shrimps with body length of approximately 6.8–9.2 cm and weight of approximately 2–5 g were used in LvSRSF2 knockdown experiments. In addition, shrimps with body length of approximately 3–5 cm and weight of approximately 0.5–1.0 g were used in survival rate experiments. Prior to experimental use, shrimp were subjected to a health screening process. Gill tissues from randomly selected individuals were used for total RNA extraction, followed by qPCR screening for White spot syndrome virus (WSSV), *Enterocytozoon hepatopenaei* (EHP), and *Vibrio parahaemolyticus* (VP_AHPND_). All tested shrimp were confirmed negative for these pathogens before inclusion in the experiments [64].

### 4.3. Phylogenetic Tree and Sequence Study of LvSRSFs

The LvSRSF sequence was obtained from the NCBI database (http://blast.ncbi.nlm.nih.gov/ accessed on 3 December 2024). Multiple sequence alignments were performed using Clustal Omega, available at https://www.ebi.ac.uk /Tools/msa/clustalo/ (accessed on 3 December 2024). A phylogenetic tree was constructed in MEGA 11.0 (http://www.megasoftware.net/ accessed on 3 December 2024) using the neighbor-joining (NJ) method with bootstrap values calculated from 1000 replicates. The Jones–Taylor–Thornton (JTT) substitution model was applied, and evolutionary rates were modeled with a Gamma distribution (G = 1.00). RRM domain predictions were made using SMART available at http://smart.embl-heidelberg.de/ (accessed on 3 December 2024).

### 4.4. Organ Distribution Analysis of LvSRSF Genes

A sample was prepared for organ distribution analysis of the LvSRSF genes by extracting total RNA from seven tissues: the hemocytes, hepatopancreas, gills, muscle, stomach, heart, and intestines of three individual shrimps. For hemocytes, the hemolymph was collected in the presence of an anticoagulant solution (anticoagulant-modified Alsever’s solution or MAS solution). The MAS solution consisted of 27 mM sodium citrate, 336 mM NaCl, 115 mM glucose, and 9 mM EDTA with a pH of 7.0 [65]. Hemocytes were obtained through centrifugation at 800× *g* and 4 °C for 10 min.

The total RNA from samples of each organ of a shrimp was extracted using the Tissue Total RNA Purification Mini Kit (Favorgen, Ping Tung, Taiwan) according to the manufacturer’s instructions. The experiments were performed in triplicate. The amount of RNA was determined using a Nanodrop 2000 Spectrophotometer (Thermo Scientific.,Vilnius, Lithuania). The quality of the RNA was assessed using agarose gel electrophoresis with visualization by staining using RedSafe Nucleic Acid Staining Solution (iNtRON)., Seongnam, Republic of Korea. A total of 1 µg of RNA samples was digested with 1 U of DNase I (Thermo Scientific.,Vilnius, Lithuania) to remove any contaminating DNA.

The cDNA was synthesized using the RevertAid First Strand cDNA Synthesis Kit by following the manufacturer’s procedure (Thermo Scientific.,Vilnius, Lithuania). The cDNA was stored at −20 °C until use in experiments. qPCR was performed to determine the expression levels of various LvSRSFs in different tissues using three technical sample replicates. Each specific LvSRSF primer isoform used to perform qRT-PCR was designed to have a product size of approximately 50–300 bp (Table 1). The elongation factor 1-alpha (EF-1α) gene was used as an internal control.

The qPCR reaction contained THUNDERBIRD^TM^ Next SYBRTM qPCR Mix (5 µL) (TOYOBO, Tokyo, Japan), 100 ng of cDNA (1 µL), 0.1 µL of 10 µM of the forward and reverse primers, and distilled water, which was added to achieve 10 µL. The CFX96 Touch Real-time PCR System (Bio-Rad Laboratories, Hercules, CA, USA) was used to perform qPCR under the following conditions: 40 cycles of amplification (95 °C for 15 s and the specific annealing temperature for each primer (Table 1) for 30 s) after an initial denaturation of 95 °C for 3 min. The relative expression level of various LvSRSFs in different tissues was calculated based on the 2^−ΔΔCT^ method [66].

### 4.5. LvSRSF Response Following WSSV Infection Using qPCR

Hemocytes play a crucial role in the shrimps’ innate immune response by eliminating foreign substances through processes such as phagocytosis, encapsulation, and nodule formation [3]. Additionally, they combat pathogens and facilitate cell repair by producing reactive oxygen species (ROS), antimicrobial peptides, immune enzymes, and various other immune factors [4,5]. During WSSV infection, WSSV134 and the hemocyte homeostasis-associated protein of *P. monodon* (*Pm*HHAP) cooperate to control apoptosis and play essential roles in host–pathogen interactions [6]. Studies on fibrinogen-related proteins (FREPs) in *L. vannamei* have demonstrated that the expression of the *LvFREP* gene is restricted to hemocytes. The expression of the *LvFREP* gene in shrimp hemocytes was specifically triggered by a *Vibrio* infection and showed no response to White spot syndrome virus (WSSV). Additionally, *LvFREP* transcripts were observed early in fertilized eggs, indicating that this immune-related gene may play a role in antimicrobial defense during shrimp development [7].

For this reason, we selected only the isoforms of *LvSRSFs* that were most highly expressed compared to other tissues and performed the experiment to investigate the expression levels of *LvSRSFs* at different time points after WSSV infection in only hemocytes.

The expression levels of LvSRSFs were investigated at different time points after WSSV infection using the isoforms of LvSRSFs that have substantially higher expression levels in hemocytes than in other tissues. The total RNA of hemocytes at 0, 6, 24, 48, and 72 h after WSSV infection was extracted using the Tissue Total RNA Purification Mini Kit. A total of 1 µg of RNA from each sample was treated with 1 U of DNase I to eliminate contaminating DNA, followed by cDNA synthesis using the RevertAid First Strand cDNA Synthesis Kit. The cDNA of hemocytes at 0, 6, 24, 48, and 72 h after WSSV infection was analyzed by qPCR with specific LvSRSF primer targets according to Section 2.4. Expression levels of LvSRSFs targeted at different times after WSSV infection were quantified using three replicates of three shrimp pooled samples.

### 4.6. Double-Stranded RNA (dsRNA) Preparation

SRSF isoform 2 of *L. vannamei* (LvSRSF2) (XM_027373369) exhibited increased expression in hemocyte samples following WSSV infection and was suppressed using dsRNA specifically designed to target it (dsLvSRSF2). The dsLvSRSF2 was designed based on positions 80–310, including an RRM domain segment. The forward primer was designed to include the restriction enzyme *Xba*I at the 5′ end, and the reverse primer was designed to include the restriction enzyme *Xho*I at the 5′ end.

The cDNA of *L. vannamei* hemocytes was used to produce templates specific for dsLvSRSF2 using PCR with Taq DNA polymerase (Vivantis, Selangor, Malaysia). The LvSRSF2 fragment was amplified using the forward and reverse primers specified for LvSRSF2 in Table 1. The PCR products were purified using the FavorPrep Gel/PCR Purification Micro Kit (Favorgen, Ping Tung, Taiwan). The LvSRSF2 product was ligated into the pTG19-T vector (Vivantis), and its accuracy was confirmed by sequencing. The recombinant LvSRSF2-pTG19-T was digested using the restriction enzymes *Xba*I and *Xho*I and then cloned into the L4440 vector (Novagen) using *Escherichia coli* HT115 as the host. The negative control included dsRNA that specifically targeted the green fluorescent protein (dsGFP).

Starter cultures of *E. coli* HT115 with recombinant dsLvSRSF2 and dsGFP were incubated in 2×YT medium at 37 °C and 250 rpm for 16–18 h. These starter cultures were used to inoculate in 2×YT medium at 1% of the total desired culture volume. The cultures were incubated at 37 °C with shaking at 200 rpm until the optical density (O.D.) at 600 nm reached approximately 0.5–0.6. Then, 1 M isopropyl β-D-thiogalactoside (IPTG) was added into the culture to achieve a final concentration of 1 mM, and the culture was incubated at 37 °C with shaking at 250 rpm for 4 h.

After 4 h of IPTG induction, the cell pellet was collected and resuspended in 70% ethanol in RNase-free 1× PBS at a ratio of 10 mL per 100 mL of the total culture volume. The cell suspension was incubated at room temperature for 15 min, followed by centrifugation at 8000× *g* at 4 °C for 15 min to collect the pellet. The pellet was resuspended in 150 mM RNase-free NaCl at a ratio of 2 mL per 100 mL of the total culture volume. The suspension was gently inverted for 1 h. The suspension was centrifuged at 10,000× *g* for 10 min at 4 °C. The supernatant was then adjusted to a final concentration of 500 mM RNase-free NaCl.

Single-stranded RNA was removed from the solution at 37 °C for 30 min using the final concentration of RNase A, which was 20 µg/mL. Solution that had been pretreated with RNase A was supplemented with GENEzol Reagent (Geneaid, New Taipei City, Taiwan) at a ratio of 1 volume of GENEzol Reagent to 1 volume of the solution. Subsequently, dsGFP or dsLvSRSF2 was extracted using the RNA extraction protocol of the GENEzol Reagent. dsGFP or dsLvSRSF2 was dissolved in DI water treated with DEPC at a ratio of 1:10,000 of the total volume of culture used for dsRNA production. The quality of dsRNA was confirmed by performing 1.2% (*w*/*v*) agarose gel electrophoresis with UV detection, staining with RedSafe Nucleic Acid Staining Solution, and quantification with a UV spectrophotometer.

### 4.7. Knockdown of LvSRSF2 Expression In Vivo by Double-Stranded RNA

LvSRSF2 was knocked down to investigate its role in the immune response to WSSV infection in white shrimp. The dsRNA was diluted to a concentration of 0.50 µg/µL using 0.85% NaCl, and 10 µg of dsRNA/g body weight was injected into the shrimp. This concentration was preliminarily tested in our laboratory.

Four main groups of nine shrimps each were examined: group 1 received only dsLvSRSF2, group 2 received only dsGFP, group 3 received dsLvSRSF2 followed by WSSV injection 24 h after dsLvSRSF2 injection, and group 4 received dsGFP followed by WSSV injection 24 h after dsGFP injection. Each group had three replicates of three shrimp pooled. The shrimp were briefly placed on ice for 30 s to induce unconsciousness. Subsequently, dsLvSRSF2 and dsGFP were injected into the shrimp, and then they were carefully returned to the water tank. The preparation of WSSV, its concentration, and the volume used are described in Section 4.2.

After injection with dsRNA at 48 and 72 h, hemolymph samples were collected, total RNA was extracted, and cDNA was synthesized using the method described in Section 2.4. The cDNA was used to analyze the expression levels of LvSRSF2, circRNAs, and linear RNAs (liRNAs) of the Anillin, Alpha2, Calpain, Hemocytin, Nephrin, and Toll genes by qPCR, and EF-1α was used as the internal control (Table 1). CircRNAs and liRNAs primer sequences were designed or obtained based on the results of transcriptome data analysis (Limkul et al., 2023) [4]. The qPCR was performed using the CFX96 Touch™ Real-Time PCR System with the conditions described in Section 4.4.

### 4.8. Mortality Assays and Quantification of WSSV Copy Number Following LvSRSF2 Knockdown and WSSV Infection

The survival rate was also monitored after separating the shrimp into six groups. Groups 1 and 2 received 10 µg/g of 0.85% NaCl, groups 3 and 4 received 10 µg/g of dsGFP, and groups 5 and 6 received 10 µg/g of dsLvSRSF2. Each group contained a total of 20 shrimps. After 24 h, WSSV was administered intramuscularly to shrimp groups 2, 4, and 6. The preparation of WSSV, its concentration, and the volume used are described in Section 4.2. Survival rates were determined every 12 h for 9 d following injection.

Gills were collected from three individual shrimp samples in groups 2, 4, and 6, and DNA was isolated using the FavorPrep™ Tissue Genomic DNA Extraction Mini Kit (Favorgen, Ping Tung, Taiwan) as a template. The amount of DNA was determined using a Nanodrop 2000 Spectrophotometer (Thermo Scientific.,Vilnius, Lithuania). The CFX96 Touch Real-time PCR System (Bio-Rad Laboratories, Hercules, CA, USA) was used to quantify WSSV copies at 24 h following WSSV injection with a VP-28-specific primer (U2Bio, Seoul, Republic of Korea) (Table 1) [67]. The reaction and conditions of qPCR are described in Section 4.4. The recombinant plasmid containing a conserved region of the VP28 gene of WSSV was used to prepare the standard curve.

### 4.9. The miRNAs-circRNAs Network and the Expression of circRNAs and Their Immune Response to WSSV Infection

To investigate the potential interaction networks, miRNAs, and circRNAs during WSSV infection in shrimp hemocytes, interaction networks were constructed based on differentially expressed miRNAs identified during WSSV challenge [30]. miRNA target prediction was conducted using CU-Mir software (developed in-house) (http://shrimp-irn.org/mirtarget/index.php, accessed on 27 April 2025) by comparing miRNA sequences against transcriptome data; percentage of sequence complementarity was calculated based on the number of percent complimentary with a cut-off set at 40%.

To confirm the expression of circRNAs and their immune response to WSSV infection. The WSSV challenge was conducted, and hemolymph samples were collected at 0, 6, 24, and 48 h hpi. Total RNA was isolated using the Tissue Total RNA Purification Mini Kit (Favorgen, Ping Tung, Taiwan). For gene expression analysis, reverse transcription was carried out using the RevertAid First Strand cDNA Synthesis Kit (Thermo Scientific, Vilnius, Lithuania), and quantitative PCR was performed using the qPCR analysis was performed using the 2×Taq THUNDERBIRD^TM^ Next SYBR qPCR (TOYOBO, Tokyo, Japan) with a specific primer (Table 1). The gene expression was quantified using the 2^−ΔΔCt^ method and normalization to elongation factor-1 alpha (EF-1α). All experiments were carried out in triplicate [4].

### 4.10. siRNA-Induced circRNA Silencing

To selectively inhibit circ-Hemocytin expression, siRNAs were specifically designed to target the back-splice junction of the circ-Hemocytin. The siRNAs were synthesized by GenePharma with (5′-3′): si-circHemocytin UGGCGCAUCCUGCUUGAUUTT and a scrambled CUCGCUACGAUUGUGUCUGTT. Each shrimp received an intramuscular injection of 100 µL of siRNA solution, which was prepared in 0.85% (*w*/*v*) NaCl at a final concentration of 6.25 µM. 24 h following the injection, shrimp were challenged with WSSV and injected intramuscularly with 100 μL of a 10^−5^ diluted viral stock. At Gill, samples were collected at 48 h hpi [59]. Total RNA was isolated using the Tissue Total RNA Purification Mini Kit (Favorgen, Ping Tung, Taiwan). For gene expression analysis, reverse transcription was carried out using the RevertAid First Strand cDNA Synthesis Kit (Thermo Scientific, Vilnius, Lithuania), and quantitative PCR was performed using the qPCR analysis was performed using the 2×Taq THUNDERBIRD Next SYBR qPCR (TOYOBO, Tokyo, Japan).

### 4.11. Statistical Analysis

All experiments were carried out in triplicate, and the results are reported using averages ± standard deviations (SDs). The results of the mortality assays are presented as the survival rate percentage ± the standard error (SE). One-way ANOVA was used to assess differences between samples in the experiments on organ distribution analysis of LvSRSF genes, the response of LvSRSF2 to WSSV infection using qPCR, and quantification of WSSV copy number in shrimp after LvSRSF2 knockdown and WSSV infection. A *t*-test was used to analyze the differences between samples in the experiment on LvSRSF2 knockdown in vivo using dsRNA. For mortality assays, simple survival analysis (Kaplan–Meier) was used to evaluate the statistical differences between samples. A *p*-value less than 0.05 indicated that the differences were statistically significant. GraphPad Prism (version 9.3.0) was used for statistical analysis.

## 5. Conclusions

In summary, this study demonstrated the identification of SRSFs from *L. vannamei*. In addition, LvSRSF2 is significantly overexpressed in hemocytes and upregulated during WSSV infection. Then, LvSRSF2 silencing increased circRNA expression, reduced WSSV replication, and prolonged shrimp survival during infection. Our findings could provide a potential molecular target for developing effective strategies to mitigate the impacts of WSSV in shrimp aquaculture

## Figures and Tables

**Table 1:** Primer sequences for qPCR analysis and cloning ds*LvSRSF2* that were used in this study.

Primer Name	Primer Sequences (5′ → 3′)	Annealing Temperature
F-*LvAlpha2* Circ R-*LvAlpha2* Circ	CACCATTACGCCCACTAGC TGTAAGGGTCCTCCACTAACTG	57 °C
F-*LvAlpha2* Li R-*LvAlpha2* Li	TCATAGCCCGCGGTAAG TGTAAGGGTCCTCCACTAAC	60 °C
F-*LvAnillin* Circ R-*LvAnillin* Circ	GGGCAGCAGCCTTTGATTC GCAGCGGGTAGCTTTCTTG	60 °C
F-*LvAnillin* Li R-*LvAnillin* Li	ACCTGAGCGCCAACAAAG TCCTGCAACAGTGTCCATC	60 °C
F-*LvCalpain* Circ R-*LvCalpain* Circ	TGAGAGTGTTCTCGGAGAAG TTCCGGAGTCCATACACTG	60 °C
F-*LvCalpain* Li R-*LvCalpain* Li	TGGCTGCCATTGCGAAC ACAAGGCCGTTGTTGCAG	57 °C
F- *EF-1α* R- *EF-1α*	CGCAAGAGCGACAACTATGA TGGCTTCAGGATACCAGTCT	60 °C
F-*LvHemocytin* Circ R-*LvHemocytin* Circ	TCGGACGCAGAGATGATTC TAGACTTCGCCGTCGATCTC	57 °C
F-*LvHemocytin* Li R-*LvHemocytin* Li	AGGCCGACTGCGAGAAGAAG TTCCTCGTGCACACGCAAG	60 °C
F-*LvNephrin* Circ R-*LvNephrin* Circ	CACGGGAATCAACCCAGCTC ACAGATCTCGACGCCGTCAG	60 °C
F-*LvNephrin* Li R-*LvNephrin* Li	AACCTACGCCGTGGAAG AGGATCCGGACTTCCATTG	60 °C
F-*LvSSRF1B* R-*LvSSRF1B*	TCATCGACCTGAAGAACCGC ACATCTCCTGCCTCCCTCAT	60 °C
F-*LvSRSF2* R-*LvSRSF2*	TTGGAGACGTGTACATCCCG CCATTCCACGCTCATTTTGCT	60 °C
F-*LvSRSF2* X1 R-*LvSRSF2* X1	TACTCGGACAATTCCAGGTCG GAGTGGCTGCCAGACCTT	60 °C
F-*LvSRSF2* X2 R-*LvSRSF2* X2	CGGGACAATTCCAGGTCG GAGTGGCTGCCAGACCTT	60 °C
F-*LvSRSF2* X4 R-*LvSRSF2* X4	CCGACGGTCGAGGTCGAG GAGTGGCTGCCAGACCTT	60 °C
F-*LvSRSF2* X5 R-*LvSRSF2* X5	TACTCGGACAATTCCAGGTCG TGTGACCTTGATCTAGAGTGC	60 °C
F-*LvSRSF2* X6 R-*LvSRSF2* X6	CGGGACAATTCCAGGTCG TGTGACCTTGATCTAGAGTGC	60 °C
F-*LvSRSF2* X7 R-*LvSRSF2* X7	CCGACGGTACTCGTCGAGG TGTGACCTTGATCTAGAGTGC	60 °C
F-*LvSRSF2* X8 R-*LvSRSF2* X8	CCGACGGTCGAGGTCGAG TGTGACCTTGATCTAGAGTGC	60 °C
F-*LvSRSF3* (XM_027370964.1) R-*LvSRSF3* (XM_027370964.1)	GTTCTCGACGGGACCGATAC TCTGGGGAATCACTTCTGCG	60 °C
F-*LvSRSF4* conserved R-*LvSRSF4* conserved	CCTAAGCTACCGTGTGGGTG GAACCCTCTTGGTGGAGGTG	60 °C
F-*LvSRSF5* R-*LvSRSF5*	GCCGTGAAAGAGACCTGGAA CGGTGAAGGTCACTTGGCTT	60 °C
F-*LvSRSF5* X1 R-*LvSRSF5* X1	GCTTGGCTTGACAGGTACGG ATCCTTGAGATCCTGCCAGC	60 °C
F-*LvSRSF5* X2 R-*LvSRSF5* X2	ATGAGTTACGCCCACGTTAG AACGGGAGTGTTGTCGATCC	60 °C
F-*LvSRSF7* conserved R-*LvSRSF7* conserved	TTTGAGGACATGCGAGATGC TGACTTCCCCGTTGATAGCTC	60 °C
F-*LvSRSF7* (XM_027216940.1) R-*LvSRSF7* (XM_027216940.1)	TTTGGCTATAAGCGACCCCC GGATCTTGATCGGCTGTGGT	60 °C
F-ds*LvSRSF2* R-ds*LvSRSF2*	CCTCTAGACAGAAGATTTGCGGCG CCCTCGAGTTGATTCACGTTGGGG	60 °C
F-*LvToll* Li R-*LvToll* Li	CGCTTCTCTGTCCTCATTTC GGTTGCCTCGAAGTTTCAG	60 °C
F-*LvToll* Circ R-*LvToll* Circ	AGGTCATCATCGCCAGCACAG ACCACCACGAGGCAAGGAAG	60 °C
F-*VP28* R-*VP28*	AAACCTCCGCATTCCTGTGA TCCGCATCTTCTTCCTTCAT	60 °C
F-STAT R-STAT	TATATCCGAATGTGCCTA ATAGTTTGTGGTGTGTTG	60 °C
F-Vago4 R-Vago4	AGCTGCTGCCCCATCATCT ATCCAATTCGTGAACTCGTCGTA	60 °C

## Data Availability

Data are available upon request to the corresponding author.

## Abbreviations

The following abbreviations are used in this manuscript:

°C	Degree Celsius
µg	Microgram
µL	Microliter
AMPs	Antimicrobial peptides
bp	Base pair
cDNA	Complementary DNA
circ-Alpha2	Circular RNA of Alpha2
circ-Anillin	Circular RNA of Anillin
circ-Calpain	Circular RNA of Calpain
circ-Hemocytin	Circular RNA of Hemocytin
circ-Nephrin	Circular RNA of Nephrin
circPLCE1	Circular phospholipase C epsilon 1
circRNAs	Circular RNAs
circ-Toll	Circular RNA of Toll
cm	Centimeter
d	Days
DECs	Differentially expressed circRNAs
DEPC	Diethyl pyrocarbonate
DI water	Deionized water
DNA	Deoxyribonucleic acid
dsGFP	dsRNA that specifically targeted the green fluorescent protein
dsLvSRSF2	dsRNA that specifically targeted the Serine/arginine splicing factor isoform 2 of *L. vannamei*
dsRNAs	Double-stranded RNA
EDTA	Ethylenediaminetetraacetic acid
EF-1α	Elongation factor 1-alpha
g	Gram
h	Hours
HBV	Hepatitis B virus
HIV-1	Human immunodeficiency virus 1
hpi	Hours post injection
HPV-16	Human papillomavirus
HSV-1	Herpes simplex virus type 1
ICP0	Infected cell polypeptide 0
ICP27	Infected cell polypeptide 27
IPTG	Isopropyl β-D-thiogalactoside
IRF	Interferon regulatory factor
JTT	The Jones–Taylor–Thornton
li-Alpha2	Linear RNA of Alpha2
li-Anillin	Linear RNA of Anillin
li-Calpain	Linear RNA of Calpain
li-Hemocytin	Linear RNA of Hemocytin
li-Nephrin	Linear RNA of Nephrin
liRNAs	Linear RNAs
li-Toll	Linear RNA of Toll
LvSRSF1B	Serine/arginine splicing factor isoform 1B of *L. vannamei*
LvSRSF2	Serine/arginine splicing factor isoform 2 of *L. vannamei*
LvSRSF2 X1	Serine/arginine splicing factor isoform 2 X1 of *L. vannamei*
LvSRSF2 X2	Serine/arginine splicing factor isoform 2 X2. of *L. vannamei*
LvSRSF2 X4	Serine/arginine splicing factor isoform 2 X4 of *L. vannamei*
LvSRSF2 X5	Serine/arginine splicing factor isoform 2 X5 of *L. vannamei*
LvSRSF2 X6	Serine/arginine splicing factor isoform 2 X6 of *L. vannamei*
LvSRSF2 X7	Serine/arginine splicing factor isoform 2 X7 of *L. vannamei*
LvSRSF2 X8	Serine/arginine splicing factor isoform 2 X8 of *L. vannamei*
LvSRSF3 X1	Serine/arginine splicing factor isoform 3 X1 of *L. vannamei*
LvSRSF3 X3	Serine/arginine splicing factor isoform 3 X3 of *L. vannamei*
LvSRSF3 X6	Serine/arginine splicing factor isoform 3 X6 of *L. vannamei*
LvSRSF4 X1	Serine/arginine splicing factor isoform 4 X1 of *L. vannamei*
LvSRSF4 X2	Serine/arginine splicing factor isoform 4 X2 of *L. vannamei*
LvSRSF4 X4	Serine/arginine splicing factor isoform 4 X4 of *L. vannamei*
LvSRSF4 X5	Serine/arginine splicing factor isoform 4 X5 of *L. vannamei*
LvSRSF5	Serine/arginine splicing factor isoform 5 of *L. vannamei*
LvSRSF5 X1	Serine/arginine splicing factor isoform 5 X1 of *L. vannamei*
LvSRSF5 X2	Serine/arginine splicing factor isoform 5 X2 of *L. vannamei*
LvSRSF7	Serine/arginine splicing factor isoform 7 of *L. vannamei*
LvSRSF7 X1	Serine/arginine splicing factor isoform 7 X1 of *L. vannamei*
LvSRSF7 X2	Serine/arginine splicing factor isoform 7 X2 of *L. vannamei*
LvSRSF7 X3	Serine/arginine splicing factor isoform 7 X3 of *L. vannamei*
LvSRSF7 X4	Serine/arginine splicing factor isoform 7 X4 of *L. vannamei*
LvSRSF7 X5	Serine/arginine splicing factor isoform 7 X5 of *L. vannamei*
LvSRSF7 X6	Serine/arginine splicing factor isoform 7 X6 of *L. vannamei*
LvSRSFs	Serine/arginine splicing factors of *L. vannamei*
M	Molar
min	Minutes
miRNAs	MicroRNAs
mL	Milliliter
mM	Micromolar
NaCl	Sodium chloride
ncRNAs	Non-coding RNAs
NJ	The neighbor-joining
nm	Nanometer
O.D.	Optical density
PAMPs	Pathogen-associated molecular patterns
PBS	Phosphate-buffered saline
PCR	Polymerase chain reaction
ppt	Parts per thousand
PRRs	Pattern recognition receptors
qPCR	Quantitative real-time PCR
qRT-PCR	Quantitative reverse transcription PCR
RBPs	RNA-binding proteins
RNA	Ribonucleic acid
RNF125	Ring Finger Protein 125
rpm	Revolutions per minute
RRM	RNA recognition motif
s	Seconds
SD	Standard deviations
SE	Standard error
siRNAs	Small interfering RNAs
SRSF1	Serine/arginine splicing factor isoform 1
SRSF10	Serine/arginine splicing factor isoform 10
SRSF2	Serine/arginine splicing factor isoform 2
SRSF3	Serine/arginine splicing factor isoform 3
SRSF5	Serine/arginine splicing factor isoform 5
SRSF9	Serine/arginine splicing factor isoform 9
SRSFs	Serine/arginine splicing factors
TLRs	Toll-like receptors
U	Unit
UV	Ultraviolet
Vif	HIV-1 viral infectivity factor
*w*/*v*	Weight per volume
WSD	White spot disease
WSSV	White spot syndrome virus
YHV	Yellow head virus
